# Supplementary material for: Cytokinin Promotes Jasmonic Acid Accumulation in the Control of Maize Leaf Growth
Source: Plants (Basel). 2023 Aug 21;12(16):3014. doi: 10.3390/plants12163014 (PMC10459232; doi:10.3390/plants12163014)
Supplement: Supplementary file 1 [file plants-12-03014-s001.zip › plants-2455793-supplementary.pdf]

Supplemental Data

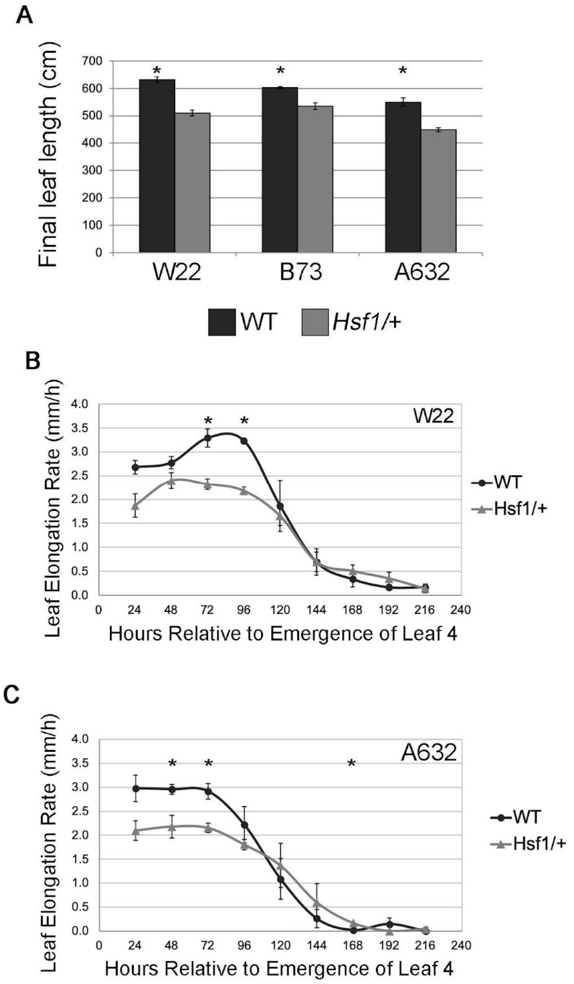

Figure S1. *Hsf1* growth in different inbred backgrounds. **(A)** Barplots of WT and *Hsf1*/+ final leaf lengths. Error bars = SE. **(B-C)** Average leaf elongation rate (LER) of leaf #4 of *Hsf1*/+ and WT siblings in the **(B)** W22, and **(C)** A632 inbred backgrounds. Asterisks mark significant differences $P < 0.05$. Error bars = SE. W22, n=3 WT, n=7 *Hsf1*/+; B73, n=5 WT, n=2 *Hsf1*/+; A632, n=4 WT, n=3 *Hsf1*/+.

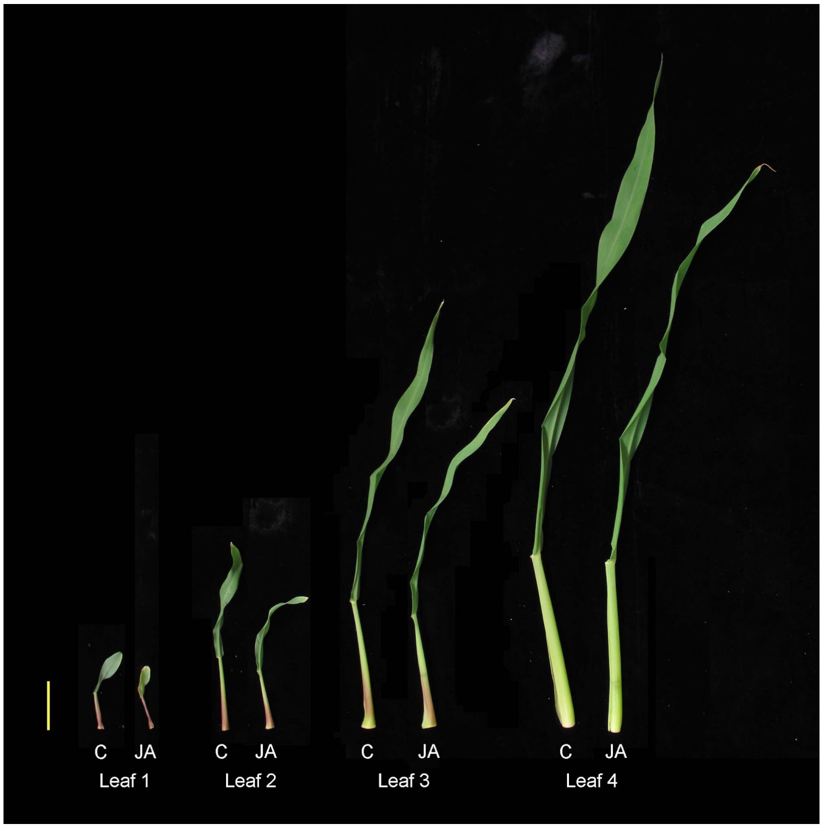

Figure S2. Comparison of control (C) and jasmonic acid (JA) treated leaves #1-4. Scale bar = 5 cm.

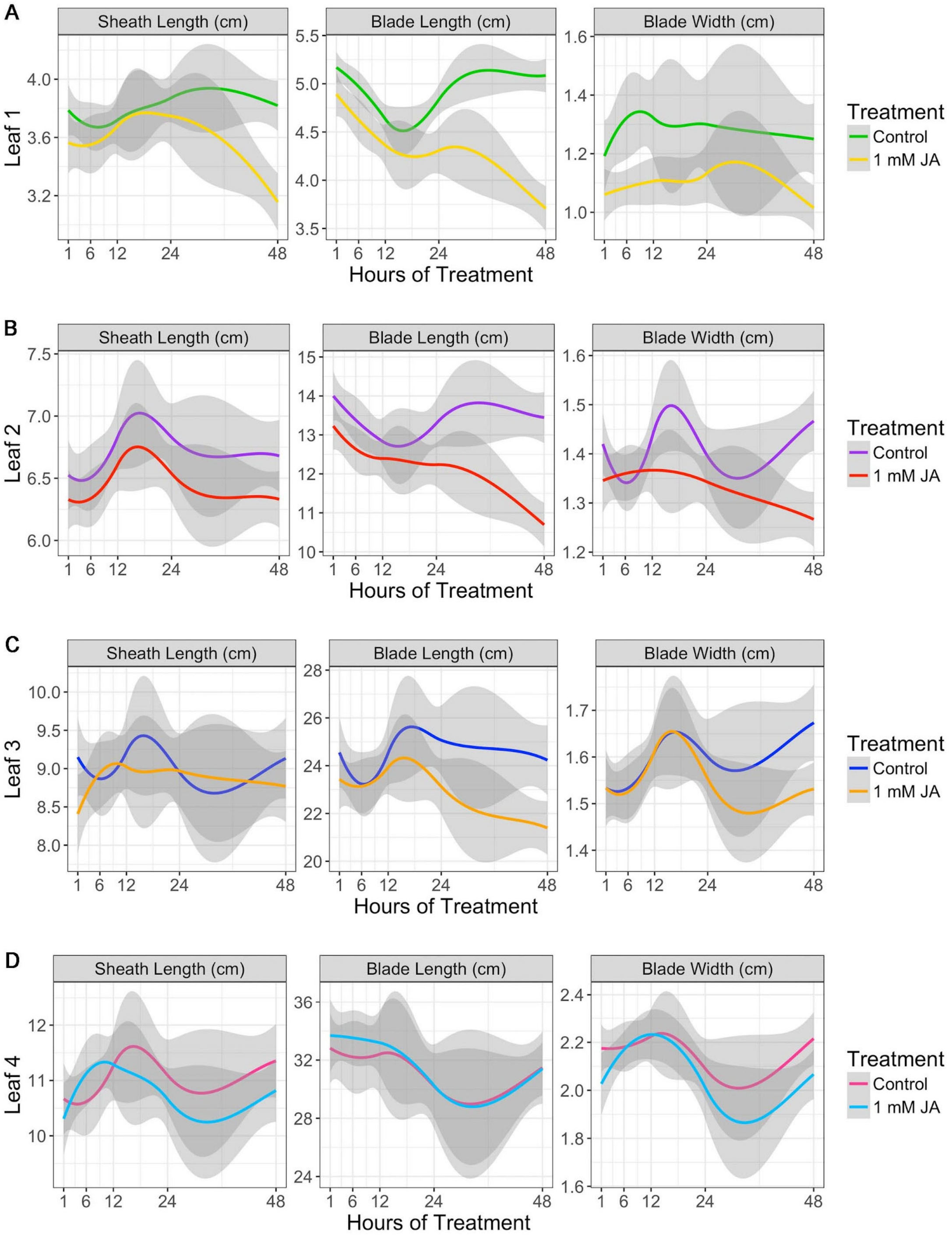

Figure S3. Final leaf measurements of B73 treated with 1 mM JA or control solution for 1, 6, 12, 24, or 48 hours. Leaf 1 (A), leaf 2 (B), leaf 3 (C), and leaf 4 (D) were measured for all plants. N ≥ 14 for each treatment group, lines are smoothed conditional means, and shaded area is the 95% confidence interval. Treatments are significant where confidence intervals do not overlap.

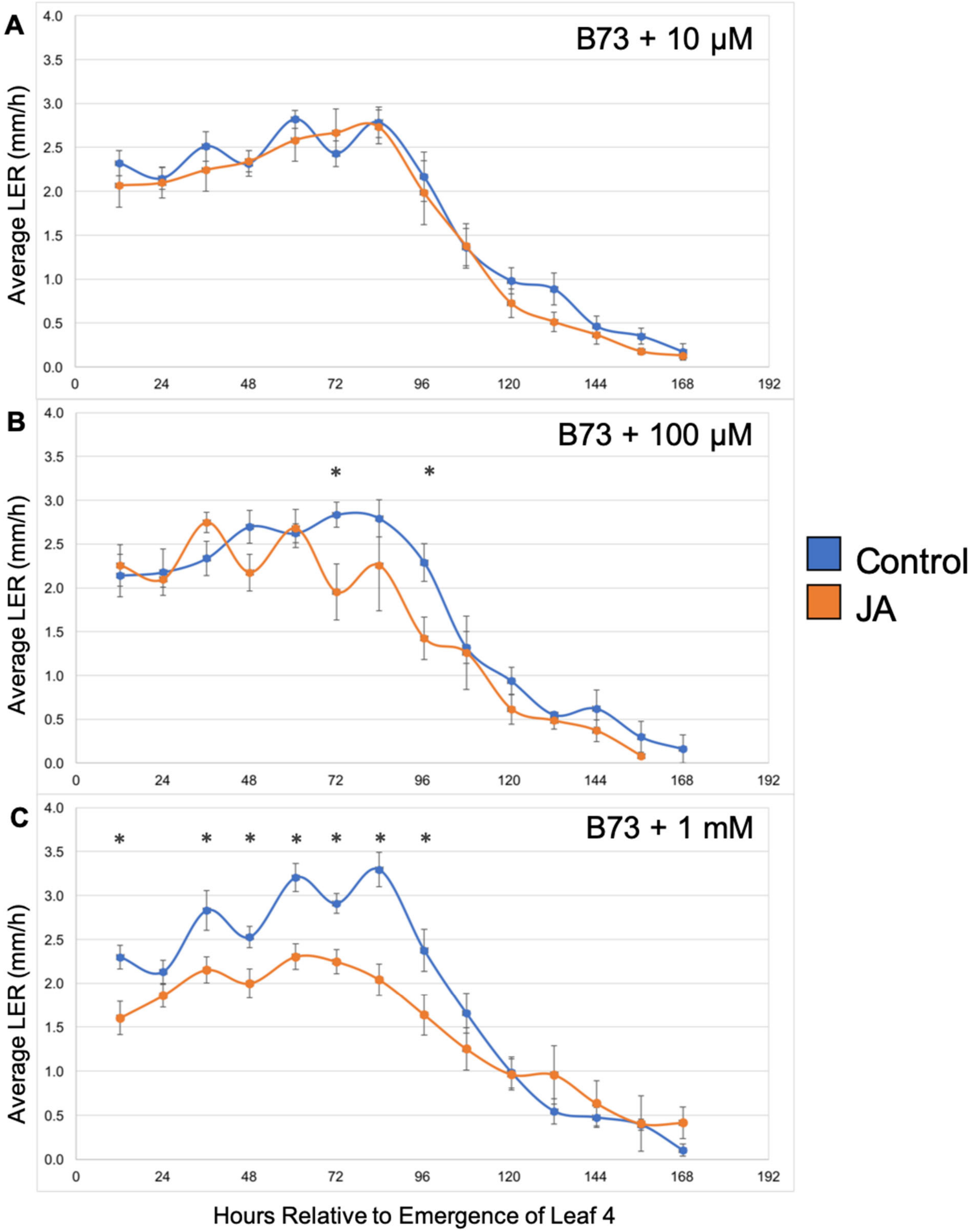

Figure S4. LER dose response to JA in B73. Leaf 4 LERs of B73 treated with (A) 10 µM, (B) 100 µM, and (C) 1 mM JA for 6 days. Significant differences $P < 0.05$ calculated by Student's t-test are marked by asterisks and error bars are SE.

Table S1. Percent leaf size reduction after exogenous 1 mM JA treatment. Percent reductions [(JA-C)/C *100] in sheath length, blade length, and blade width by leaf number. Red means significant value P < 0.05.

	Leaf 1	Leaf 2	Leaf 3	Leaf 4
Sheath Length	-23.30%	-30.90%	-21.90%	-17.80%
Blade Length	-29.90%	-28.70%	-30.50%	-26.00%
Blade Width	-18.70%	-9.30%	-15.30%	-14.30%

Table S2. Percent leaf size reduction after 48 hours of exogenous 1 mM JA treatment. Percent reductions [(JA-C)/C *100] in sheath length, blade length, and blade width by leaf number. Red means significant value P < 0.05.

	Leaf 1	Leaf 2	Leaf 3	Leaf 4
Sheath Length	-17.30%	-5.20%	-4.00%	-4.70%
Blade Length	-27.10%	-20.50%	-11.70%	-0.30%
Blade Width	-18.80%	-13.60%	-8.50%	-6.70%

Table S3. Relevant comparisons of *Hsf1*/+ and WT-sibling final leaf size percent reductions after JA treatment. (i) WT-sibling compared to *Hsf1*/+ without JA, (ii) WT-sibling with JA treatment, (iii) *Hsf1*/+ with JA treatment, (iv) WT-sibling compared to *Hsf1*/+ both treated with JA. Red means significant percent difference $P < 0.05$.

i. WT Control vs. *Hsf1*/+ Control

	Leaf 1	Leaf 2	Leaf 3	Leaf 4
Sheath Length	-22.20%	-18.40%	-16.50%	-14.50%
Blade Length	-20.20%	-21.90%	-22.80%	-13.50%
Blade Width	-22.90%	-20.60%	-21.80%	-15.20%

ii. WT Control vs. WT JA

	Leaf 1	Leaf 2	Leaf 3	Leaf 4
Sheath Length	-28.80%	-16.90%	-15.90%	-12.20%
Blade Length	-44.80%	-39.00%	-25.80%	-18.90%
Blade Width	-27.80%	-23.40%	-20.40%	-10.60%

iii. *Hsf1*/+ Control vs. *Hsf1*/+ JA

	Leaf 1	Leaf 2	Leaf 3	Leaf 4
Sheath Length	-16.50%	-15.80%	-15.00%	-7.80%
Blade Length	-38.10%	-37.40%	-25.30%	-17.50%
Blade Width	-29.00%	-9.20%	-17.60%	-13.40%

iv. WT JA vs. *Hsf1*/+ JA

	Leaf 1	Leaf 2	Leaf 3	Leaf 4
Sheath Length	-8.80%	-17.30%	-15.60%	-10.20%
Blade Length	-10.50%	-19.80%	-22.40%	-12.00%
Blade Width	-24.20%	-5.90%	-19.10%	-17.90%

Table S4. Relevant comparisons of *opr7 opr8* double mutant final leaf size percent reductions. (i) *opr7 opr8* compared to JA sufficient *opr7/opr7 OPR8/opr8* (ii) *opr7 opr8* compared to JA sufficient *opr7/opr7 OPR8/OPR8*. Red means significant percent difference P < 0.05.

**i. *opr7/opr7 opr8/opr8* vs. *opr7/opr7 OPR8/opr8***				
	**Leaf 1**	**Leaf 2**	**Leaf 3**	**Leaf 4**
Sheath Length	39.5%	22.2%	11.0%	-1.4%
Blade Length	43.0%	36.8%	22.6%	14.2%
Blade Width	-7.6%	-1.6%	10.0%	17.6%

**ii. *opr7/opr7 opr8/opr8* vs. *opr7/opr7 OPR8/OPR8***				
	**Leaf 1**	**Leaf 2**	**Leaf 3**	**Leaf 4**
Sheath Length	43.3%	21.0%	12.4%	-2.3%
Blade Length	48.2%	38.2%	24.2%	12.9%
Blade Width	-9.5%	-4.8%	9.2%	18.6%

Table S5. The list of primers used in this study.

Primer Name	Target Gene (MaizeGDB loci)	Sequence	Product (bp)	Purpose
ARV0090*	Mu-9242	AGAGAAGCCAACGCCAWCGCCTCYATTTCGTC	n/a	Genotype *opr7 opr8*
ARV0097	*OPR7* *(Zm00001d032049)*	CGACACACATGCTCAAAATCGAGA	WT = 816 *opr7* = 418/398	
ARV0098		CTCCACCAGACCATCAGATCTAGC		
ARV0099	*OPR8* *(Zm00001d050107)*	TATGGCAAGTATCCAACTCCGAGG	WT =942 *opr8* =530/412	
ARV0100		ACACGAACAATAGTCCGCCTCTTA		
ARV0143	*Ts5* *(Zm00001d049201)*	ACACGCAATGTTTTGCTGC	WT = 129 Ts5/+ = 129/138	Genotype *Ts5/+*
ARV0144		ggccgtatcttcgctggata		
ARV0129	*ABPH1* *(Zm00001d002982)*	AGGATTTCCTGCTGAAGC	174	qRT-PCR
ARV0130		GACACAGAGCTTCGGAAT		
ARV0131	*RR6* *(Zm00001d001865)*	TCATGTCATCGGAGAACGTG	245	qRT-PCR
ARV0132		TCCCCCCAAATGTTAGCTC		
ARV0113	*CKO2* (Zm00001d042148)	TTCAACCCTCCTTCCGTCTTCC	135	qRT-PCR
ARV0114		TGGGGAGCTTGGAATCAGAAGG		
ARV0177	*ts1* (Zm00001d003533)	CCCCAACAGCGTTACCATTT	191	qRT-PCR
ARV0178		CTGTTCGGACCACCAAATCA		
ARV0183	*AOS1a* (Zm00001d034184)	ACTACCTTCTGCTCCTCCCCACCCG	133	qRT-PCR
ARV0184		GCGGTATGCTAGAACGGACA		
ARV0189	*AOS2a* *(Zm00001d028282)*	GAAAACCCTGAAACCTTGGTGAAG	101	qRT-PCR
ARV0190		GAGGGCGCTACTGAAACCAC		
ARV0135	*AOC1* *(Zm00001d029594)*	TCAATTCGTGAGTGCGTGGTAG	87	qRT-PCR
ARV0136		ATCAGAAGCAGCCAAGTGAAGC		
ARV0179	*AOC2* *(Zm00001d047340)*	TCACAAACAAGGTTTACAACGGCAGC	182	qRT-PCR
ARV0182		CGACTCCTCGTAGGTCAGGTACGC		
ARV0033	*OPR7* *(Zm00001d032049)*	CACATTCCTCGGCCAGCCTA	130	qRT-PCR
ARV0034		TCGCGGCATTACCCAGATGT		
ARV0027	*OPR8* *(Zm00001d050107)*	AGTATCCAACTCCGAGGCGC	80	qRT-PCR
ARV0028		GGCGTTTATGGCAGCCTGTC		
ARV0037	*JAR1b*	CCCGGTCGCTACGTCATCTT	94	qRT-PCR

ARV0038	*(Zm00001d009714)*	CGACGAAGGCTAGGTCCAGG		
ARV0195	*JAR2b (Zm00001d039346)*	TGATGGACAGCACGAGGTTC	73	qRT-PCR
ARV0196		AAGGTGGCCGGTCTCTACAA		
ARV0011	*Ts5 (Zm00001d049201)*	CCGCGCTGACATGGTTCTTC	131	qRT-PCR
ARV0012		GTGGAGCACGCTCATCTTGC		
ARV0081	*MYC7 (Zm00001d030028)*	AACCGATGTTAGCGTTGGGTTG	138	qRT-PCR
ARV0082		AAGCCTGACTCCATTGCAAAGC		
ARV0235	*MPI (Zm00001d011080)*	ACGCCAAGAAGGTGATCCTC	106	qRT-PCR
ARV2036		AAGATGCGGACACGGTTAGG		
ARV0045	TBP1 (*Zm00001d033472*)	CCCTGGTGAACACGGACACT	243	qRT-PCR control gene
ARV0046		CTTGCCCTTTGCCCTTGTCC		

*Serves as a forward and reverse.

Table S6. Metabolite-specific parameters used in triple quad LC-MS for hormone quantification in B73 and *Hsf1*.

**Abbreviation**	**Chemical/Common Name**	**RT (min)**	**MW**	**Q1 (m/z)**	**Q3 (m/z)**
12COOH-JA-Ile	12-carboxy-jasmonic acid	5.4	339.3	352	130
12OH-JA-Ile	12-hydroxy-jasmonic acid isoluecine	5.3	353	338.3	130
12-OPDA	12-oxo-10(*Z*),15(*Z*)-phytodienoic acid	11.02	292.4	291.2	165.1
GA3	gibberelic acid-3	3.98	346.4	345.2	239.1
IAA	auxin / indole-3-acetic acid	4.98	175.18	174	130
JA	jasmonic acid	6.7	210.27	209.1	59
JA-Ile	jasmonic acid isoluecine	8.2	323.44	322.2	130
SA	salicylic acid	4.26	138.12	137	92.9
